# Barrier Function Adaptive Nonsingular Terminal Sliding Mode Control Approach for Quad-Rotor Unmanned Aerial Vehicles

**DOI:** 10.3390/s22030909

**Published:** 2022-01-25

**Authors:** Khalid A. Alattas, Omid Mofid, Abdullah K. Alanazi, Hala M. Abo-Dief, Andrzej Bartoszewicz, Mohsen Bakouri, Saleh Mobayen

**Affiliations:** 1 Department of Computer Science and Artificial Intelligence, College of Computer Science and Engineering, University of Jeddah, Jeddah 23890, Saudi Arabia; kaalattas@uj.edu.sa; 2 Future Technology Research Center, National Yunlin University of Science and Technology, Douliou 64002, Yunlin, Taiwan; D10913003@yuntech.edu.tw; 3 Department of Chemistry, Faculty of Science, Taif University, P.O. Box 11099, Taif 21944, Saudi Arabia; aalanaz4@tu.edu.sa (A.K.A.); h.abodeif@tu.edu.sa (H.M.A.-D.); 4 Institute of Automatic Control, Lodz University of Technology, 18/22 Stefanowskiego St., 90-924 Lodz, Poland; andrzej.bartoszewicz@p.lodz.pl; 5 Department of Medical Equipment Technology, College of Applied Medical Science, Majmaah University, Majmaah 11952, Saudi Arabia; 6 Department of Physics, College of Arts, Fezzan University, Traghen 71340, Libya

**Keywords:** quad-rotor system, barrier function technique, adaptive law, non-singular terminal sliding mode, matched disturbance

## Abstract

This paper proposes a barrier function adaptive non-singular terminal sliding mode controller for a six-degrees-of-freedom (6DoF) quad-rotor in the existence of matched disturbances. For this reason, a linear sliding surface according to the tracking error dynamics is proposed for the convergence of tracking errors to origin. Afterward, a novel non-singular terminal sliding surface is suggested to guarantee the finite-time reachability of the linear sliding surface to origin. Moreover, for the rejection of the matched disturbances that enter into the quad-rotor system, an adaptive control law based on barrier function is recommended to approximate the matched disturbances at any moment. The barrier function-based control technique has two valuable properties. First, this function forces the error dynamics to converge on a region near the origin in a finite time. Secondly, it can remove the increase in the adaptive gain because of the matched disturbances. Lastly, simulation results are given to demonstrate the validation of this technique.

## 1. Introduction

The small type of unmanned aerial vehicles (UAVs) is named a quad-rotor, which has received significant consideration over the past decades [1,2]. This kind of UAV has noteworthy properties, including a simple structure and easy operation [3,4]. In addition, its precise industrial applications, such as damage inspection, border patrolling, and mapping, cannot be denied [5,6]. Therefore, it is very important to design a high-performance control method for quad-rotor systems in different situations. Most designed control methods for quad-rotor systems focus on the position and attitude of the desired tracking control of the quad-rotor system, which forces the quad-rotor into the desired location [7,8,9]. Due to this, some important issues have to be considered in the control of the quad-rotor system. One of the main issues that has to be considered in the control of the quad-rotor is related to fast-tracking control of the quad-rotor. On the other hand, the quad-rotor system should track the desired location in a finite time [1,10]. For this reason, a non-singular terminal sliding mode control (TSMC) technique with a low reachability time is planned to increase the convergence rate of the states of the quad-rotor system to the origin [11,12,13,14,15]. In [16], the fast nonsingular TSMC technique is proposed for the finite-time stability of the position and attitude loops of the quad-rotor under torque disturbances. A non-singular mixed with super-twisting algorithm is proposed in [17] for the tracking control of the quad-rotor in conditions of unmodeled dynamics and external disturbances. Moreover, a second-order disturbance observer is designed for the rejection of the uncertainties and perturbations. In [18], a second-order SMC method based on the non-singular mixed with super-twisting algorithm is recommended for the attitude tracking control of the quad-rotor in conditions of exterior disturbances. In [19], a continuous non-singular TSMC tactic is used for tracking control of the quad-rotor. In order to obtain a better performance when disturbances occur, an adaptive non-singular TSMC scheme is applied.

Another issue in the design of controllers for the quad-rotor is the consideration of the matched disturbances, which always exist in practice [20,21,22,23,24,25]. The visual quadrotor tracking of an uncertain ground moving target is studied in [20] by using the neuro-adaptive integral robust control technique. In [21], reference trajectory tracking control and active disturbance rejection for UAV systems by processing the measurable outputs are proposed. However, differentiation in the signals and disturbance estimation are not required. An active disturbance rejection switching control scheme is suggested in [22] for trajectory tracking of quadrotor UAVs based on a robust differentiator. One of the methods for the rejection of the matched disturbances is the usage of the adaptive-based barrier function. This method guarantees fast convergence of the trajectories of the system when it is combined with other control methods [26,27,28]. Moreover, this method is robust against variation of the perturbations [29]. In [30], a first-order SMC approach is proposed based on adaptive control using the barrier function. This method ensures fast convergence of the trajectories in the disturbed system without knowledge of the upper bound of the disturbances. In [31], an adaptive higher-order SMC method based on the barrier function is suggested to achieve finite-time stability of system in the presence of bounded uncertainty. In [32], a barrier function-based adaptive SMC (ASMC) method is recommended for the stability of the disturbed nonlinear system. In [33], the barrier function-based adaptive feedback control scheme, with the aim of achieving stability of the spacecraft in the existence of parameter uncertainties and perturbations, is presented. So, uncertainties and perturbations are approximated using the barrier function.

According to the review of the recent articles about attitude and position tracking control of quad-rotors in the presence of matched disturbances, it can be concluded that no work has investigated the adaptive barrier function technique using the non-singular TSMC method for disturbance rejection and tracking control of the disturbed quad-rotor system. In this paper, finite-time tracking control and disturbance rejection of the quad-rotor system in the presence of matched disturbances are investigated based on the adaptive non-singular TSMC method using the barrier function theory. Therefore, the chief contributions of this work are reported as follows:

- Presentation of a linear sliding surface aiming for convergence of thee attitude and position tracking error;
- Proposition of a nonsingular terminal sliding surface as the target of fast convergence of the linear sliding surface;
- Employment of the adaptive barrier function technique for rejection of the matched disturbances that enter the quad-rotor system;
- Demonstration of finite-time tracking control of the disturbed quad-rotor system using the Lyapunov stability concept.

The remaining sections of this study are included as follows: the dynamic model of the quad-rotor system under matched disturbances is presented in Section 2. The main results related to the definition of the nonsingular terminal sliding surface are stated in Section 3. In Section 4, the adaptive barrier function non-singular terminal sliding mode control scheme is designed. The simulation outcomes are described in Section 5. Lastly, the conclusion is provided in Section 6.

## 2. Presentation of the Dynamical Model of the Quad-Rotor

In this section, first, a dynamical model related to the position and attitude of the 6DoF quad-rotor system is considered. Then, for the simplicity of the control process, the considered dynamical model is expressed in the state-space formulation for the appearance of the matched disturbances.

The dynamical model of the quad-rotor with 6DoF was obtained using the Newton–Euler formula in several works [34,35,36]. Presume x, y, z and ϕ, θ, ψ are the variables of the position/attitude of the quad-rotor system; hence, the dynamical equation relevant to the position/attitude of 6DoF quad-rotor is taken as:

(1) $$\begin{array}{l} \ddot{x}\left(t\right)=\frac{1}{M}\left[-{K_{fd}}_{xx}\dot{x}\left(t\right)+u_{z}\left(t\right)u_{x}\left(t\right)\right],\\ \ddot{y}\left(t\right)=\frac{1}{M}\left[-{K_{fd}}_{yy}\dot{y}\left(t\right)+u_{z}\left(t\right)u_{y}\left(t\right)\right],\\ \ddot{z}\left(t\right)=\frac{1}{M}\left[-{K_{fd}}_{zz}\dot{z}\left(t\right)+\left(cos\left(\phi \right)cos\left(\theta \right)\right)u_{z}\left(t\right)\right]-g,\\ \ddot{\phi }\left(t\right)=\frac{1}{I_{xx}}\left[\left(I_{yy}-I_{zz}\right)\dot{\psi }\left(t\right)\dot{\theta }\left(t\right)-{K_{fa}}_{xx}{\dot{\phi }}^{2}\left(t\right)-J_{r}\bar{\Omega }\dot{\theta }\left(t\right)+Du_{\phi }\left(t\right)\right],\\ \ddot{\theta }\left(t\right)=\frac{1}{I_{yy}}\left[\left(I_{zz}-I_{xx}\right)\dot{\psi }\left(t\right)\dot{\phi }\left(t\right)-{K_{fa}}_{yy}{\dot{\theta }}^{2}\left(t\right)+J_{r}\bar{\Omega }\dot{\phi }\left(t\right)+Du_{\theta }\left(t\right)\right],\\ \ddot{\psi }\left(t\right)=\frac{1}{I_{zz}}\left[\left(I_{xx}-I_{yy}\right)\dot{\phi }\left(t\right)\dot{\theta }\left(t\right)-{K_{fa}}_{zz}{\dot{\psi }}^{2}\left(t\right)+C_{D}u_{\psi }\left(t\right)\right]. \end{array}$$

where $u_{x}$, $u_{y}$, $u_{z}$, $u_{\phi }$, $u_{\theta }$, and $u_{\psi }$ are considered as control inputs and $\bar{\Omega }=w_{1}-w_{2}+w_{3}-w_{4}$.

**Remark** **1.** *The quad-rotor system is an underactuated system with four control inputs*$u_{z}$, $u_{\phi }$, $u_{\theta }$, and $u_{\psi }$. Thus, $u_{x}$ and $u_{y}$ *are considered as auxiliary control inputs for the conversion of the quad-rotor model into the six degrees of freedom structure.*

**Remark** **2.** *The used parameters in the presentation of the dynamic model of the quad-rotor system are introduced in Table 1.*

When the control of the quad-rotor is operated with the velocity of the motor, the following relations between the control inputs and velocities exist:

(2) $$\left\{\begin{array}{c} u_{z}\left(t\right)=K_{p}\left(w_{1}^{2}+w_{2}^{2}+w_{3}^{2}+w_{4}^{2}\right)\\ u_{\phi }\left(t\right)=-K_{p}w_{1}^{2}+K_{p}w_{3}^{2}\\ u_{\theta }\left(t\right)=-K_{p}w_{2}^{2}+K_{p}w_{4}^{2}\\ u_{\psi }\left(t\right)=C_{d}\left(w_{1}^{2}-w_{2}^{2}+w_{3}^{2}-w_{4}^{2}\right) \end{array}\right.$$

To simplify the dynamical equation, the following variables are defined as:

(3) $$\left\{\begin{array}{c} \rho_{1}=\frac{-{K_{fd}}_{xx}}{M},\;\rho_{2}=\frac{-{K_{fd}}_{yy}}{M},\;\rho_{3}=\frac{-{K_{fd}}_{zz}}{M}\\ \rho_{4}=\frac{I_{yy}-I_{zz}}{I_{xx}},\;\rho_{5}=\frac{-{K_{fa}}_{xx}}{I_{xx}},\;\rho_{6}=-\frac{J_{r}}{I_{xx}}\\ \rho_{7}=\frac{I_{zz}-I_{xx}}{I_{yy}},\;\rho_{8}=\frac{-{K_{fa}}_{yy}}{I_{yy}},\;\rho_{9}=\frac{J_{r}}{I_{yy}}\\ \rho_{10}=\frac{I_{xx}-I_{yy}}{I_{zz}},\;\rho_{11}=\frac{-{K_{fa}}_{zz}}{I_{zz}}\\ \varrho_{1}=\frac{D}{I_{xx}},\;\varrho_{2}=\frac{D}{I_{yy}},\;\varrho_{3}=\frac{C_{D}}{I_{zz}} \end{array}\right.$$

Hence, Equation (1) is re-expressed as:

(4) $$\left\{\begin{array}{c} \ddot{x}\left(t\right)=\rho_{1}\dot{x}\left(t\right)+\frac{u_{zz}}{M}u_{x}\left(t\right)\\ \ddot{y}\left(t\right)=\rho_{2}\dot{y}\left(t\right)+\frac{u_{zz}}{M}u_{y}\left(t\right)\\ \ddot{z}\left(t\right)=\rho_{3}\dot{z}\left(t\right)-g+\frac{\left(cos\left(\phi \right)cos\left(\theta \right)\right)}{M}u_{z}\left(t\right)\\ \ddot{\phi }\left(t\right)=\rho_{4}\dot{\psi }\left(t\right)\dot{\theta }\left(t\right)+\rho_{5}{\dot{\phi }}^{2}\left(t\right)+\rho_{6}\bar{\Omega }\dot{\theta }\left(t\right)+\varrho_{1}u_{\phi }\left(t\right)\\ \ddot{\theta }\left(t\right)=\rho_{7}\dot{\psi }\left(t\right)\dot{\phi }\left(t\right)+\rho_{8}{\dot{\theta }}^{2}\left(t\right)+\rho_{9}\bar{\Omega }\dot{\phi }\left(t\right)+\varrho_{2}u_{\theta }\left(t\right)\\ \ddot{\psi }\left(t\right)=\rho_{10}\dot{\phi }\left(t\right)\dot{\theta }\left(t\right)+\rho_{11}{\dot{\psi }}^{2}\left(t\right)+\varrho_{3}u_{\psi }\left(t\right) \end{array}\right.$$

Now, the new state variables are defined as:

(5) $$\left\{\begin{array}{c} z_{1}\left(t\right)=x\left(t\right),\;z_{2}\left(t\right)=\dot{x}\left(t\right)\\ z_{3}\left(t\right)=y\left(t\right),\;z_{4}\left(t\right)=\dot{y}\left(t\right)\\ z_{5}\left(t\right)=z\left(t\right),\;z_{6}\left(t\right)=\dot{z}\left(t\right)\\ z_{7}\left(t\right)=\phi \left(t\right),\;z_{8}\left(t\right)=\dot{\phi }\left(t\right)\\ z_{9}\left(t\right)=\theta \left(t\right),\;z_{10}\left(t\right)=\dot{\theta }\left(t\right)\\ z_{11}\left(t\right)=\psi \left(t\right),\;z_{12}\left(t\right)=\dot{\psi }\left(t\right) \end{array}\right.$$

Hence, Equation (4) is considered in the state-space form under matched disturbances $\Delta_{i}\left(t\right),\;\forall i=x,y,z,\phi ,\theta ,\psi$ with an unknown bound $Q_{i}\in R$, i.e., ${\left|\Delta_{i}\left(t\right)\right|}_{max}\leq Q_{i}$ as:

(6) $$\left\{\begin{array}{c} {\dot{z}}_{1}\left(t\right)=z_{2}\left(t\right)\\ {\dot{z}}_{2}\left(t\right)=\rho_{1}z_{2}\left(t\right)+\frac{u_{z}\left(t\right)}{M}u_{x}\left(t\right)+\Delta_{x}\left(t\right)\\ {\dot{z}}_{3}\left(t\right)=z_{4}\left(t\right)\\ \rho_{10}=\frac{I_{xx}-I_{yy}}{I_{zz}},\rho_{11}=\frac{-{K_{fa}}_{zz}}{I_{zz}}\\ {\dot{z}}_{5}\left(t\right)=z_{6}\left(t\right)\\ {\dot{z}}_{6}\left(t\right)=\rho_{3}z_{6}\left(t\right)-g+\frac{\left(cos\left(\phi \right)cos\left(\theta \right)\right)}{M}u_{z}\left(t\right)+\Delta_{z}\left(t\right)\\ {\dot{z}}_{7}\left(t\right)=z_{8}\left(t\right)\\ {\dot{z}}_{8}\left(t\right)=\rho_{4}z_{12}\left(t\right)z_{10}\left(t\right)+\rho_{5}z_{8}{}^{2}\left(t\right)+\rho_{6}\bar{\Omega }z_{10}\left(t\right)+\varrho_{1}u_{\phi }\left(t\right)+\Delta_{\phi }\left(t\right)\\ {\dot{z}}_{9}\left(t\right)=z_{10}\left(t\right)\\ {\dot{z}}_{10}\left(t\right)=\rho_{7}z_{12}\left(t\right)z_{8}\left(t\right)+\rho_{8}z_{10}{}^{2}\left(t\right)+\rho_{9}\bar{\Omega }z_{8}\left(t\right)+\varrho_{2}u_{\theta }\left(t\right)+\Delta_{\theta }\left(t\right)\\ {\dot{z}}_{11}\left(t\right)=z_{12}\left(t\right)\\ {\dot{z}}_{12}\left(t\right)=\rho_{10}z_{8}\left(t\right)z_{10}\left(t\right)+\rho_{11}z_{12}{}^{2}\left(t\right)+\varrho_{3}u_{\psi }\left(t\right)+\Delta_{\psi }\left(t\right) \end{array}\right.$$

## 3. Main Results

The main control objective in tracking control of the quad-rotor is the design of the controller, which forces the position/attitude of the quad-rotor to track the desired position. For this reason, the stability of the tracking errors among the position and attitude and their desired values is the key problem in control of the quad-rotor system. Thus, in this study, a linear sliding surface is used on the target of the tracking errors’ stabilization. Then, a non-singular terminal sliding surface is recommended to achieve stability of the linear sliding surface in a finite time.

Now, the tracking errors are assumed as:

(7) $$\left\{\begin{array}{c} E_{x}\left(t\right)=z_{1}\left(t\right)-x_{d}\left(t\right)\\ E_{y}\left(t\right)=z_{3}\left(t\right)-y_{d}\left(t\right)\\ E_{z}\left(t\right)=z_{5}\left(t\right)-z_{d}\left(t\right)\\ E_{\phi }\left(t\right)=z_{7}\left(t\right)-\phi_{d}\left(t\right)\\ E_{\theta }\left(t\right)=z_{9}\left(t\right)-\theta_{d}\left(t\right)\\ E_{\psi }\left(t\right)=z_{11}\left(t\right)-\psi_{d}\left(t\right) \end{array}\right.$$

where $x_{d}\left(t\right)$, $y_{d}\left(t\right)$, $z_{d}\left(t\right)$, $\phi_{d}\left(t\right)$, $\theta_{d}\left(t\right)$, and $\psi_{d}\left(t\right)$ denote the desired values of the position/attitude of the quad-rotor.

In order to stabilize the tracking errors (Equation (7)), the linear sliding functions are defined as:

(8) $$s_{i}\left(t\right)=C_{1}E_{i}\left(t\right)+C_{2}{\dot{E}}_{i}\left(t\right),\;\left(\forall i=x,y,z,\phi ,\theta ,\psi \right)$$

where $C_{1}\in R^{m\times \left(n-m\right)}$ and $C_{2}\in R^{m\times m}$ are the constant matrices with $rank\left(C_{2}\right)=m$. When the linear sliding function is obtained, i.e., $s_{i}\left(t\right)=0$, Equation (8) provides:

(9) $${\dot{E}}_{i}\left(t\right)=-C_{2}^{-1}C_{1}E_{i}\left(t\right).$$

From Equations (6), (7) and (9), the sliding dynamics are:

(10) $$\left\{\begin{array}{l} {\dot{z}}_{1}\left(t\right)=-C_{2}^{-1}C_{1}\left(z_{1}\left(t\right)-x_{d}\left(t\right)\right)+{\dot{x}}_{d}\left(t\right)\\ {\dot{z}}_{3}\left(t\right)=-C_{2}^{-1}C_{1}\left(z_{3}\left(t\right)-y_{d}\left(t\right)\right)+{\dot{y}}_{d}\left(t\right)\\ {\dot{z}}_{5}\left(t\right)=-C_{2}^{-1}C_{1}\left(z_{5}\left(t\right)-z_{d}\left(t\right)\right)+{\dot{z}}_{d}\left(t\right)\\ {\dot{z}}_{7}\left(t\right)=-C_{2}^{-1}C_{1}\left(z_{7}\left(t\right)-\phi_{d}\left(t\right)\right)+{\dot{\phi }}_{d}\left(t\right)\\ {\dot{z}}_{9}\left(t\right)=-C_{2}^{-1}C_{1}\left(z_{9}\left(t\right)-\theta_{d}\left(t\right)\right)+{\dot{\theta }}_{d}\left(t\right)\\ {\dot{z}}_{11}\left(t\right)=-C_{2}^{-1}C_{1}\left(z_{11}\left(t\right)-\psi_{d}\left(t\right)\right)+{\dot{\psi }}_{d}\left(t\right) \end{array}\right.$$

In order to obtain convergence of the sliding function $s_{i}\left(t\right)$ to the origin in a finite time, the nonsingular terminal sliding surfaces are presented by:

(11) $$\sigma_{i}\left(t\right)=s_{i}\left(t\right)+\alpha_{i}\int_{0}^{t}s_{i}{\left(\tau \right)}^{\frac{p_{i}}{q_{i}}}d\tau ,$$

where $p_{i}$ and $q_{i}$ denote two odd integers, $\alpha_{i}>0$, $1<p_{i}/q_{i}<2$. Differentiating Equation (8) gives:

(12) $${\ddot{E}}_{i}\left(t\right)=-C_{2}^{-1}C_{1}{\dot{E}}_{i}\left(t\right)$$

From (5) and (6), we obtain:

(13) $$\left\{\begin{array}{c} {\dot{z}}_{2}\left(t\right)=-C_{2}^{-1}C_{1}{\dot{E}}_{x}\left(t\right)+{\ddot{x}}_{d}\left(t\right)\\ {\dot{z}}_{4}\left(t\right)=-C_{2}^{-1}C_{1}{\dot{E}}_{y}\left(t\right)+{\ddot{y}}_{d}\left(t\right)\\ {\dot{z}}_{6}\left(t\right)=-C_{2}^{-1}C_{1}{\dot{E}}_{z}\left(t\right)+{\ddot{z}}_{d}\left(t\right)\\ {\dot{z}}_{8}\left(t\right)=-C_{2}^{-1}C_{1}{\dot{E}}_{\phi }\left(t\right)+{\ddot{\phi }}_{d}\left(t\right)\\ {\dot{z}}_{10}\left(t\right)=-C_{2}^{-1}C_{1}{\dot{E}}_{\theta }\left(t\right)+{\ddot{\theta }}_{d}\left(t\right)\\ {\dot{z}}_{12}\left(t\right)=-C_{2}^{-1}C_{1}{\dot{E}}_{\psi }\left(t\right)+{\ddot{\psi }}_{d}\left(t\right) \end{array}\right.$$

where equating the right-hand sides of Equations (5) and (12), the equivalent controllers are found as:

(14) $$\left\{\begin{array}{c} {u_{x}}_{eq}\left(t\right)=-\frac{M}{u_{z}\left(t\right)}\left(\rho_{1}z_{2}\left(t\right)-{\ddot{x}}_{d}\left(t\right)+C_{2}^{-1}C_{1}{\dot{E}}_{x}\left(t\right)\right)\\ {u_{z}}_{eq}\left(t\right)=-\frac{M}{\cos\left(\phi \right)\cos\left(\theta \right)}\left(\rho_{3}z_{6}\left(t\right)-g-{\ddot{z}}_{d}\left(t\right)+C_{2}^{-1}C_{1}{\dot{E}}_{z}\left(t\right)\right)\\ {u_{y}}_{eq}\left(t\right)=-\frac{M}{u_{z}\left(t\right)}\left(\rho_{2}z_{4}\left(t\right)-{\ddot{y}}_{d}\left(t\right)+C_{2}^{-1}C_{1}{\dot{E}}_{y}\left(t\right)\right)\\ {u_{\phi }}_{eq}\left(t\right)=-\frac{1}{\varrho_{1}}\left(\rho_{4}z_{12}\left(t\right)z_{10}\left(t\right)+\rho_{5}z_{8}{}^{2}\left(t\right)+\rho_{6}\bar{\Omega }z_{10}\left(t\right)-{\ddot{\phi }}_{d}\left(t\right)+C_{2}^{-1}C_{1}{\dot{E}}_{\phi }\left(t\right)\right)\\ {u_{\theta }}_{eq}\left(t\right)=-\frac{1}{\varrho_{2}}\left(\rho_{7}\dot{\psi }\left(t\right)\dot{\phi }\left(t\right)+\rho_{8}{\dot{\theta }}^{2}\left(t\right)+\rho_{9}\bar{\Omega }\dot{\phi }\left(t\right)-{\ddot{\theta }}_{d}\left(t\right)+C_{2}^{-1}C_{1}{\dot{E}}_{\theta }\left(t\right)\right)\\ {u_{\psi }}_{eq}\left(t\right)=-\frac{1}{\varrho_{3}}\left(\rho_{10}z_{8}\left(t\right)z_{10}\left(t\right)+\rho_{11}z_{12}{}^{2}\left(t\right)-{\ddot{\psi }}_{d}\left(t\right)+C_{2}^{-1}C_{1}{\dot{E}}_{\psi }\left(t\right)\right) \end{array}\right.$$

**Theorem** **1.** *Consider the disturbed quad-rotor system (6). Using the control input as:*

(15) $$u_{i}\left(t\right)={u_{i}}_{eq}\left(t\right)+{u_{i}}_{N}\left(t\right)$$

*with the equivalent controller (14) and the discontinuous control law as:*

(16) $$\left\{\begin{array}{c} {u_{x}}_{N}\left(t\right)=-\frac{M}{u_{z}\left(t\right)}\left(\left(Q_{x}+\mu_{x}\right)\mathrm{sgn}(\sigma_{x}\left(t\right)\right)+\alpha_{x}s_{x}{\left(t\right)}^{\frac{p_{x}}{q_{y}}})\\ {u_{y}}_{N}\left(t\right)=-\frac{M}{u_{z}\left(t\right)}\left(\left(Q_{y}+\mu_{y}\right)\mathrm{sgn}(\sigma_{y}\left(t\right)\right)+\alpha_{y}s_{y}{\left(t\right)}^{\frac{p_{y}}{q_{y}}})\\ {u_{z}}_{N}\left(t\right)=-\frac{M}{\left(\cos\left(\phi \right)\cos\left(\theta \right)\right)}\left(\left(Q_{z}+\mu_{z}\right)\mathrm{sgn}(\sigma_{z}\left(t\right)\right)+\alpha_{z}s_{z}{\left(t\right)}^{\frac{p_{z}}{q_{z}}})\\ {u_{\phi }}_{N}\left(t\right)=-\frac{1}{\varrho_{1}}\left(\left(Q_{\phi }+\mu_{\phi }\right)\mathrm{sgn}(\sigma_{\phi }\left(t\right)\right)+\alpha_{\phi }s_{\phi }{\left(t\right)}^{\frac{p_{\phi }}{q_{\phi }}})\\ {u_{\theta }}_{N}\left(t\right)=-\frac{1}{\varrho_{2}}\left(\left(Q_{\theta }+\mu_{\theta }\right)\mathrm{sgn}(\sigma_{\theta }\left(t\right)\right)+\alpha_{\theta }s_{\theta }{\left(t\right)}^{\frac{p_{\theta }}{q_{\theta }}})\\ {u_{\psi }}_{N}\left(t\right)=-\frac{1}{\varrho_{3}}\left(\left(Q_{\psi }+\mu_{\psi }\right)\mathrm{sgn}(\sigma_{\psi }\left(t\right)\right)+\alpha_{\psi }s_{\psi }{\left(t\right)}^{\frac{p_{\psi }}{q_{\psi }}}) \end{array}\right.$$

*where* $\mu_{i}>0$ *and* ${\left|\Delta_{i}\left(t\right)\right|}_{max}\leq Q_{i}$ *. Therefore, nonsingular terminal sliding surfaces converge to zero in finite time. Then, the states of the system (6) are forced to move from the initial conditions to the nonsingular terminal sliding surface (11) and stay on it. So, attitude and position tracking control of the quad-rotor is accomplished under matched disturbances appropriately.*

**Proof.**  The time-derivative of the nonsingular terminal sliding surfaces is:

(17) $${\dot{\sigma }}_{i}\left(t\right)={\dot{s}}_{i}\left(t\right)+\alpha_{i}s_{i}{\left(\tau \right)}^{\frac{p_{i}}{q_{i}}}.$$

Consider the Lyapunov function:

(18) $$V_{i}\left(\sigma_{i}\left(t\right)\right)=\frac{1}{2}\sigma_{i}{}^{T}\left(t\right)\sigma_{i}\left(t\right)$$

where the time-derivative of Equation (18) is given by:

(19) $${\dot{V}}_{i}\left(\sigma_{i}\left(t\right)\right)=\sigma_{i}{}^{T}\left(t\right){\dot{\sigma }}_{i}\left(t\right)$$

Substituting. Equation (17) into (19) and considering Equations (7) and (8), we obtain:

(20) $${\dot{V}}_{1}{}_{i}\left(\sigma_{i}\left(t\right)\right)=\sigma_{i}{}^{T}\left(t\right)\left(C_{2}{}^{-1}C_{1}{\dot{E}}_{i}\left(t\right)+{\ddot{E}}_{i}\left(t\right)+\alpha_{i}s_{i}{\left(t\right)}^{\frac{p_{i}}{q_{i}}}\right)$$

Substituting Equation (6) into (20), we obtain:

(21) $$\begin{array}{c} {\dot{V}}_{1}{}_{x}\left(\sigma_{x}\left(t\right)\right)=\sigma_{x}{}^{T}\left(t\right)(C_{2}{}^{-1}C_{1}{\dot{E}}_{x}\left(t\right)+\left(\rho_{1}z_{2}\left(t\right)+\frac{u_{z}\left(t\right)}{M}u_{x}\left(t\right)+\Delta_{x}\left(t\right)-{\ddot{x}}_{d}\left(t\right)\right)\\ +\alpha_{x}s_{x}{\left(t\right)}^{\frac{p_{x}}{q_{x}}}),\\ {\dot{V}}_{1}{}_{y}\left(\sigma_{y}\left(t\right)\right)=\sigma_{y}{}^{T}\left(t\right)(C_{2}{}^{-1}C_{1}{\dot{E}}_{y}\left(t\right)+\left(\rho_{2}z_{4}\left(t\right)+\frac{u_{z}\left(t\right)}{M}u_{y}\left(t\right)+\Delta_{y}\left(t\right)-{\ddot{y}}_{d}\left(t\right)\right)\\ +\alpha_{y}s_{y}{\left(t\right)}^{\frac{p_{y}}{q_{y}}}),\\ {\dot{V}}_{1}{}_{z}\left(\sigma_{z}\left(t\right)\right)=\sigma_{z}{}^{T}\left(t\right)(C_{2}{}^{-1}C_{1}{\dot{E}}_{z}\left(t\right)+(\rho_{3}z_{6}\left(t\right)-g+\frac{\left(\cos\left(\phi \right)\cos\left(\theta \right)\right)}{M}u_{z}\left(t\right)\\ +\Delta_{z}\left(t\right)-{\ddot{z}}_{d}\left(t\right))+\alpha_{z}s_{z}{\left(t\right)}^{\frac{p_{z}}{q_{z}}}),\\ {\dot{V}}_{1}{}_{\phi }\left(\sigma_{\phi }\left(t\right)\right)=\sigma_{\phi }{}^{T}\left(t\right)(C_{2}{}^{-1}C_{1}{\dot{E}}_{\phi }\left(t\right)+(\rho_{4}z_{12}\left(t\right)z_{10}\left(t\right)+\rho_{5}z_{8}{}^{2}\left(t\right)+\rho_{6}\bar{\Omega }z_{10}\left(t\right)\\ +\varrho_{1}u_{\phi }\left(t\right)+\Delta_{\phi }\left(t\right)-{\ddot{\phi }}_{d}\left(t\right))+\alpha_{\phi }s_{\phi }{\left(t\right)}^{\frac{p_{\phi }}{q_{\phi }}}),\\ {\dot{V}}_{1}{}_{\theta }\left(\sigma_{\theta }\left(t\right)\right)=\sigma_{\theta }{}^{T}\left(t\right)(C_{2}{}^{-1}C_{1}{\dot{E}}_{\theta }\left(t\right)+(\rho_{7}z_{12}\left(t\right)z_{8}\left(t\right)+\rho_{8}z_{10}{}^{2}\left(t\right)+\rho_{9}\bar{\Omega }z_{8}\left(t\right)\\ +\varrho_{2}u_{\theta }\left(t\right)+\Delta_{\theta }\left(t\right)-{\ddot{\theta }}_{d}\left(t\right))+\alpha_{\theta }s_{\theta }{\left(t\right)}^{\frac{p_{\theta }}{q_{\theta }}}),\\ {\dot{V}}_{1}{}_{\psi }\left(\sigma_{\psi }\left(t\right)\right)=\sigma_{\psi }{}^{T}\left(t\right)(C_{2}{}^{-1}C_{1}{\dot{E}}_{\psi }\left(t\right)+(\rho_{10}z_{8}\left(t\right)z_{10}\left(t\right)+\rho_{11}z_{12}{}^{2}\left(t\right)\\ +\varrho_{3}u_{\psi }\left(t\right)+\Delta_{\psi }\left(t\right)-{\ddot{\psi }}_{d}\left(t\right))+\alpha_{\psi }s_{\psi }{\left(t\right)}^{\frac{p_{\psi }}{q_{\psi }}}) \end{array}$$

Using Equations (14)–(16), we obtain:

(22) $$\begin{array}{ll} {\dot{V}}_{1}{}_{i}\left(\sigma_{i}\left(t\right)\right) & =\sigma_{i}{}^{T}\left(t\right)\left(\left(Q_{i}+\mu_{i}\right)sgn(\sigma_{i}\left(t\right)-\Delta_{i}\left(t\right)\right)\\ & \leq -\left(Q_{i}-\left|\Delta_{i}\left(t\right)\right|\right)\left|\sigma_{i}\left(t\right)\right|-\mu_{i}\left|\sigma_{i}\left(t\right)\right|\\ & \leq -\mu_{i}\left|\sigma_{i}\left(t\right)\right|\leq -2^{1/2}\mu_{i}V_{1}{{}_{i}}^{1/2}\left(\sigma_{i}\left(t\right)\right). \end{array}$$

Finally, the proof is completed. □

From Theorem 1, the nonsingular terminal sliding surfaces (11) reach zero in finite time. Then, we obtain:

(23) $${\dot{\sigma }}_{i}s_{i}\left(t\right)+\alpha_{i}\int_{0}^{t}s_{i}{\left(\tau \right)}^{\frac{p_{i}}{q_{i}}}d\tau =0$$

where defining ${ℑ}_{i}=\int_{0}^{t}s_{i}\left(\tau \right)d\tau$ and ${\dot{ℑ}}_{i}=s_{i}\left(t\right)$, Equation (23) provides:

(24) $$\frac{{\dot{ℑ}}_{i}}{{ℑ}_{i}{}^{\frac{p_{i}}{q_{i}}}}=-\alpha_{i}$$

Now, integrating both sides of Equation (24) from 0 to $t_{s}$, we obtain:

(25) $$\int_{{ℑ}_{i}\left(0\right)}^{{ℑ}_{i}\left(t_{s}\right)}{ℑ}_{i}{}^{\frac{p_{i}}{q_{i}}}{\dot{ℑ}}_{i}d{ℑ}_{i}=-\int_{0}^{t_{s}}\alpha_{i}dt=-\alpha_{i}t_{s},$$

which results in:

(26) $$t_{s}=\frac{{ℑ}_{i}{\left(0\right)}^{1-\frac{p_{i}}{q_{i}}}}{\alpha_{i}\left(1-\frac{p_{i}}{q_{i}}\right)}=\frac{s_{i}{\left(0\right)}^{1-\frac{p_{i}}{q_{i}}}}{\alpha_{i}\left(1-\frac{p_{i}}{q_{i}}\right)}.$$

## 4. Adaptive Barrier Function Technique

In this section, to overcome the matched disturbances that enter the dynamical model of the system, the adaptive control procedure is adopted. However, the adaptive control gains are changed by variations in the matched disturbances. To eliminate the increase or decrease in the adaptive control gain, an adaptive controller using the barrier function is proposed in this paper. Using the proposed barrier adaptive sliding mode control scheme, the matched disturbance can be approximated effectively, and the closed-loop system can become stable in a finite time. Using the barrier function, the nonlinear control law can be designed as:

(27) $$\left\{\begin{array}{c} u_{xN}(t)=-\frac{M}{\left(u_{z}(t\right)}(({\hat{Q}}_{x}+\mu_{x})sgn(\sigma_{x}(t))+\alpha_{x}s_{x}{\left(t\right)}^{\frac{p_{x}}{q_{y}}})\\ u_{yN}(t)=-\frac{M}{\left(u_{z}(t\right)}(({\hat{Q}}_{y}+\mu_{y})sgn(\sigma_{y}(t))+\alpha_{y}s_{y}{\left(t\right)}^{\frac{py}{q_{y}}})\\ u_{xN}(t)=-\frac{M}{\left(cos(\phi )cos(\theta )\right)}(({\hat{Q}}_{z}+\mu_{z})sgn(\sigma_{z}(t))+\alpha_{z}s_{z}{\left(t\right)}^{\frac{p_{z}}{q_{z}}})\\ u_{\phi N}(t)=-\frac{1}{\varrho_{1}}(({\hat{Q}}_{\phi }+\mu_{\phi })sgn(\sigma_{\phi }(t))+\alpha_{\phi }s_{\phi }{\left(t\right)}^{{}^{\frac{p_{\phi }}{q_{\phi }}}})\\ u_{\theta N}(t)=-\frac{1}{\varrho_{2}}(({\hat{Q}}_{{}_{\theta }}+\mu_{{}_{\theta }})sgn(\sigma_{{}_{\theta }}(t))+\alpha_{{}_{\theta }}s_{\theta }{\left(t\right)}^{{}^{\frac{p_{\theta }}{q_{\theta }}}})\\ u_{\psi N}(t)=-\frac{1}{\varrho_{3}}(({\hat{Q}}_{\psi }+\mu_{\psi })sgn(\sigma_{\psi }(t))+\alpha_{\psi }s_{\psi }{\left(t\right)}^{{}^{\frac{p\psi }{q_{\psi }}}}) \end{array}\right.$$

with:

(28) $${\hat{Q}}_{i}\left(t\right)=\left\{\begin{array}{lll} Q_{i}{}_{a}\left(t\right) & if & 0<t\leq \bar{t}\\ Q_{i}{}_{psb}\left(t\right) & if & t>\bar{t} \end{array}\right.$$

where $\bar{t}$ is the time in which the states converge to the neighborhood $\varepsilon_{i}$ of the terminal sliding mode surface $\sigma_{i}\left(t\right)$. The adaptive-tuning law and the (positive-semi-definite) barrier function are given by:

(29) $${\dot{Q}}_{i}{}_{a}\left(t\right)=\ell_{i}\left|\sigma_{i}\left(t\right)\right|$$

(30) $$Q_{i}{}_{psb}=\frac{\left|\sigma_{i}\left(t\right)\right|}{\varepsilon_{i}-\left|\sigma_{i}\left(t\right)\right|}$$

where $\ell_{i},\varepsilon_{i}>0$. According to the adaptation law (28), the control gain ${\hat{Q}}_{i}\left(t\right)$ is increased until the states reach the neighborhood $\varepsilon_{i}$ of the terminal sliding surface $\sigma_{i}\left(t\right)$ at time $\bar{t}$. Then, for instants after $\bar{t}$, the adaptive control gain ${\hat{Q}}_{i}\left(t\right)$ switches to the positive-semi-definite barrier function, which can decrease the convergence region and maintain the system states in this region. The stability of the system is verified in two conditions: (a) $0<t\leq \bar{t}$, (b) $t>\bar{t}$.

*Condition (a):* $0<t\leq \bar{t}$

**Theorem** **2.** *Consider the disturbed quad-rotor system (6). Using the adaptive control law (29) with the equivalent controller (14) and the discontinuous controller (27) considering ${\hat{Q}}_{i}\left(t\right)=Q_{i}{}_{a}\left(t\right)$, then the system state trajectories reach the neighborhood $\varepsilon_{i}$ of the terminal sliding surface in finite time.*

**Proof.**  Construct the Lyapunov candidate functional as:

(31) $${V_{2}}_{i}\left(\sigma_{i}\left(t\right),{Q_{i}}_{a}\left(t\right)\right)=0.5\left(\sigma_{i}{}^{T}\left(t\right)\sigma_{i}\left(t\right)+\varphi_{i}{}^{-1}{\left({Q_{i}}_{a}\left(t\right)-Q_{i}\right)}^{2}\right)$$

where $\varphi_{i}>0$, and $Q_{i}$ is a positive unknown constant. The time-derivative of ${V_{2}}_{i}\left(\sigma_{i}\left(t\right),{Q_{i}}_{a}\left(t\right)\right)$ is:

(32) $${{\dot{V}}_{2}}_{i}\left(\sigma_{i}\left(t\right),{Q_{i}}_{a}\left(t\right)\right)=\sigma_{i}{}^{T}\left(t\right){\dot{\sigma }}_{i}\left(t\right)+\varphi_{i}{}^{-1}\left({{\dot{Q}}_{i}}_{a}\left(t\right)-Q_{i}\right){\dot{Q}}_{i}{}_{a}\left(t\right)$$

Substituting Equation (17) and the adaptation laws (29) into the above equation with consideration of Equations (6)–(8), we obtain:

(33) $${{\dot{V}}_{2}}_{i}\left(\sigma_{x}\left(t\right),{Q_{x}}_{a}\left(t\right)\right)=\sigma_{x}{}^{T}\left(t\right)(C_{2}{}^{-1}C_{1}{\dot{E}}_{x}\left(t\right)+(\rho_{1}z_{2}\left(t\right)\;+\frac{u_{z}\left(t\right)}{M}u_{x}\left(t\right)+\Delta_{x}\left(t\right)-{\ddot{x}}_{d}\left(t\right))+\alpha_{x}s_{x}{\left(t\right)}^{\frac{p_{x}}{q_{x}}})+\ell_{x}\varphi_{x}{}^{-1}\left({Q_{x}}_{a}\left(t\right)-Q_{x}\right)\left|\sigma_{i}\left(t\right)\right|,\;{\dot{V}}_{2}{}_{y}\left(\sigma_{y}\left(t\right),{Q_{y}}_{a}\left(t\right)\right)=\sigma_{y}{}^{T}\left(t\right)(C_{2}{}^{-1}C_{1}{\dot{E}}_{y}\left(t\right)+(\rho_{2}z_{4}\left(t\right)\;+\frac{u_{z}\left(t\right)}{M}u_{y}\left(t\right)+\Delta_{y}\left(t\right)-{\ddot{y}}_{d}\left(t\right))+\alpha_{y}s_{y}{\left(t\right)}^{\frac{p_{y}}{q_{y}}})+\ell_{y}\varphi_{y}{}^{-1}\left({Q_{y}}_{a}\left(t\right)-Q_{y}\right)\left|\sigma_{y}\left(t\right)\right|,\;{\dot{V}}_{2}{}_{z}\left(\sigma_{z}\left(t\right),{Q_{z}}_{a}\left(t\right)\right)=\sigma_{z}{}^{T}\left(t\right)(C_{2}{}^{-1}C_{1}{\dot{E}}_{z}\left(t\right)+(\rho_{3}z_{6}\left(t\right)-g+\frac{\cos\left(\phi \right)\cos\left(\theta \right)}{M}u_{z}\left(t\right)\;+\Delta_{z}\left(t\right)-{\ddot{z}}_{d}\left(t\right))+\alpha_{z}s_{z}{\left(t\right)}^{\frac{p_{z}}{q_{z}}})+\ell_{z}\varphi_{z}{}^{-1}\left({Q_{z}}_{a}\left(t\right)-Q_{z}\right)\left|\sigma_{z}\left(t\right)\right|,\;{\dot{V}}_{2}{}_{\phi }\left(\sigma_{\phi }\left(t\right),{Q_{\phi }}_{a}\left(t\right)\right)=\sigma_{\phi }{}^{T}\left(t\right)(C_{2}{}^{-1}C_{1}{\dot{E}}_{\phi }\left(t\right)+(\rho_{4}z_{12}\left(t\right)z_{10}\left(t\right)+\rho_{5}z_{8}{}^{2}\left(t\right)\;+\rho_{6}\bar{\Omega }z_{10}\left(t\right)+\varrho_{1}u_{\phi }\left(t\right)+\Delta_{\phi }\left(t\right)-{\ddot{\phi }}_{d}\left(t\right))+\alpha_{\phi }s_{\phi }{\left(t\right)}^{\frac{p_{\phi }}{q_{\phi }}})+\ell_{\phi }\varphi_{\phi }{}^{-1}\left({Q_{\phi }}_{a}\left(t\right)-Q_{\phi }\right)\left|\sigma_{\phi }\left(t\right)\right|,\;{{\dot{V}}_{2}}_{\theta }\left(\sigma_{\theta }\left(t\right),{Q_{\theta }}_{a}\left(t\right)\right)=\sigma_{\theta }{}^{T}\left(t\right)(C_{2}{}^{-1}C_{1}{\dot{E}}_{\phi }\left(t\right)+(\rho_{7}\dot{\psi }\left(t\right)\dot{\phi }\left(t\right)+\rho_{8}{\dot{\theta }}^{2}\left(t\right)\;+\rho_{9}\bar{\Omega }\dot{\phi }\left(t\right)+\varrho_{2}u_{\theta }\left(t\right)+\Delta_{\theta }\left(t\right)-{\ddot{\theta }}_{d}\left(t\right))+\alpha_{\phi }s_{\phi }{\left(t\right)}^{\frac{p_{\phi }}{q_{\phi }}})+\ell_{\theta }\varphi_{\theta }{}^{-1}\left({Q_{\theta }}_{a}\left(t\right)-Q_{\theta }\right)\left|\sigma_{\theta }\left(t\right)\right|,\;{{\dot{V}}_{2}}_{\sigma }\left(\sigma_{\psi }\left(t\right),{Q_{\psi }}_{a}\left(t\right)\right)=\sigma_{\psi }{}^{T}\left(t\right)(C_{2}{}^{-1}C_{1}{\dot{E}}_{\psi }\left(t\right)+(\rho_{10}z_{8}\left(t\right)z_{10}\left(t\right)+\rho_{11}z_{12}{}^{2}\left(t\right)\;+\varrho_{3}u_{\psi }\left(t\right)+\Delta_{\psi }\left(t\right)-{\ddot{\psi }}_{d}\left(t\right))+\alpha_{\psi }s_{\psi }{\left(t\right)}^{\frac{p_{\psi }}{q_{\psi }}})+\ell_{\psi }\varphi_{\psi }{}^{-1}\left({Q_{\psi }}_{a}\left(t\right)-Q_{\psi }\right)\left|\sigma_{\psi }\left(t\right)\right|.$$

Substituting the equivalent controller (13) and discontinuous controller (27) in the above equation, we obtain:

(34) $$\begin{array}{c} {{\dot{V}}_{2}}_{i}\left(\sigma_{i}\left(t\right),{Q_{i}}_{a}\left(t\right)\right)=-\sigma_{i}{}^{T}\left(t\right)\left(\left({Q_{i}}_{a}\left(t\right)+\mu_{i}\right)\mathrm{sgn}(\sigma_{i}\left(t\right)\right)-\Delta_{i}\left(t\right))\\ +\ell_{i}\varphi_{i}{}^{-1}\left({Q_{i}}_{a}\left(t\right)-Q_{i}\right)\left|\sigma_{i}\left(t\right)\right|\\ \leq -\mu_{i}\left|\sigma_{i}\left(t\right)\right|+\left|\sigma_{i}\left(t\right)\right|\left|\Delta_{i}\left(t\right)\right|-\sigma_{i}{}^{T}\left(t\right){Q_{i}}_{a}\left(t\right)\mathrm{sgn}(\sigma_{i}\left(t\right))\\ +\ell_{i}\varphi_{i}{}^{-1}\left({Q_{i}}_{a}\left(t\right)-Q_{i}\right)\left|\sigma_{i}\left(t\right)\right|\\ \leq \left|\sigma_{i}\left(t\right)\right|\left|\Delta_{i}\left(t\right)\right|-{Q_{i}}_{a}\left(t\right)\left|\sigma_{i}\left(t\right)\right|+\ell_{i}\varphi_{i}{}^{-1}\left({Q_{i}}_{a}\left(t\right)-Q_{i}\right)\left|\sigma_{i}\left(t\right)\right|\\ \leq \left|\sigma_{i}\left(t\right)\right|\left|\Delta_{i}\left(t\right)\right|-{Q_{i}}_{a}\left(t\right)\left|\sigma_{i}\left(t\right)\right|+\ell_{i}\varphi_{i}{}^{-1}\left({Q_{i}}_{a}\left(t\right)-Q_{i}\right)\left|\sigma_{i}\left(t\right)\right|\\ +Q_{i}\left|\sigma_{i}\left(t\right)\right|-Q_{i}\left|\sigma_{i}\left(t\right)\right|\\ \leq -\left(Q_{i}-\left|\Delta_{i}\left(t\right)\right|\right)\left|\sigma_{i}\left(t\right)\right|-\left(1-\ell_{i}\varphi_{i}{}^{-1}\right)\left({Q_{i}}_{a}\left(t\right)-Q_{i}\right)\left|\sigma_{i}\left(t\right)\right| \end{array}$$

where, because $Q_{i}-\left|\Delta_{i}\left(t\right)\right|>0$ and $\ell_{i}\varphi_{i}{}^{-1}<1$, Equation (34) is written as:

(35) $$\begin{array}{c} {{\dot{V}}_{2}}_{i}\left(\sigma_{i}\left(t\right),{Q_{i}}_{a}\left(t\right)\right)\leq -\sqrt{2}\left(Q_{i}-\left|\Delta_{i}\left(t\right)\right|\right)\frac{\left|\sigma_{i}\left(t\right)\right|}{\sqrt{2}}-\sqrt{2\varphi_{i}}(1\\ -\ell_{i}\varphi_{i}{}^{-1})\left|\sigma_{i}\left(t\right)\right|\frac{\left({Q_{i}}_{a}\left(t\right)-Q_{i}\right)}{\sqrt{2\varphi_{i}}}\\ \leq -\min\left\{\sqrt{2}\left(Q_{i}-\left|\Delta_{i}\left(t\right)\right|\right),\sqrt{2\varphi_{i}}\left(1-\ell_{i}\varphi_{i}{}^{-1}\right)\left|\sigma_{i}\left(t\right)\right|\right\}\left(\frac{\left|\sigma_{i}\left(t\right)\right|}{\sqrt{2}}+\frac{\left|{Q_{i}}_{a}\left(t\right)-Q_{i}\right|}{\sqrt{2\varphi_{i}}}\right)\\ \leq -\Psi_{i}V_{2}{{}_{i}}^{\frac{1}{2}}\left(\sigma_{i}\left(t\right),{Q_{i}}_{a}\left(t\right)\right).s \end{array}$$

where $\Psi_{i}=\min\left\{\sqrt{2}\left(Q_{i}-\left|\Delta_{i}\left(t\right)\right|\right),\sqrt{2\varphi_{i}}\left(1-\ell_{i}\varphi_{i}{}^{-1}\right)\left|\sigma_{i}\left(t\right)\right|\right\}$. □

*Condition (b):* $t>\bar{t}$

**Theorem** **3.** *For the disturbed quad-rotor system (6), using the adaptive control law (30) with the equivalent controller (14) and the discontinuous controller (27) considering* ${\hat{Q}}_{i}={Q_{i}}_{psb}$ *, then the states of the system reach the convergence region* $\left|\sigma_{i}\left(t\right)\right|\leq \varepsilon_{i}$ *in finite time.*

**Proof.**  Construct the Lyapunov candidate functional as:

(36) $${V_{3}}_{i}\left(\sigma_{i}\left(t\right),{Q_{i}}_{psb}\left(t\right)\right)=0.5\left(\sigma_{i}{}^{T}\left(t\right)\sigma_{i}\left(t\right)+{\left({Q_{i}}_{psb}\left(t\right)-{Q_{i}}_{psb}\left(0\right)\right)}^{2}\right)$$

The time-derivative of ${V_{3}}_{i}\left(\sigma_{i}\left(t\right),{Q_{i}}_{psb}\left(t\right)\right)$ is:

(37) $${{\dot{V}}_{3}}_{i}\left(\sigma_{i}\left(t\right),{Q_{i}}_{psb}\left(t\right)\right)=\sigma_{i}{}^{T}\left(t\right){\dot{\sigma }}_{i}\left(t\right)+\left({Q_{i}}_{psb}\left(t\right)-{Q_{i}}_{psb}\left(0\right)\right){{\dot{Q}}_{i}}_{psb}\left(t\right)$$

where ${Q_{i}}_{psb}\left(0\right)=0$ leads to:

(38) $${{\dot{V}}_{3}}_{i}\left(\sigma_{i}\left(t\right),{Q_{i}}_{psb}\left(t\right)\right)=\sigma_{i}{}^{T}\left(t\right){\dot{\sigma }}_{i}\left(t\right)+{Q_{i}}_{psb}\left(t\right){{\dot{Q}}_{i}}_{psb}\left(t\right)$$

Substituting (17) into the above equation with consideration of Equations (6)–(8), we obtain:

(39) $${{\dot{V}}_{2}}_{x}\left(\sigma_{x}\left(t\right),{Q_{x}}_{a}\left(t\right)\right)=\sigma_{x}{}^{T}\left(t\right)(C_{2}{}^{-1}C_{1}{\dot{E}}_{x}\left(t\right)+(\rho_{1}z_{2}\left(t\right)\;+\frac{u_{z}\left(t\right)}{M}u_{x}\left(t\right)+\Delta_{x}\left(t\right)-{\ddot{x}}_{d}\left(t\right))+\alpha_{x}s_{x}{\left(t\right)}^{\frac{p_{x}}{q_{x}}})+{Q_{x}}_{psb}\left(t\right){{\dot{Q}}_{x}}_{psb}\left(t\right),\;{{\dot{V}}_{2}}_{y}\left(\sigma_{y}\left(t\right),{Q_{y}}_{a}\left(t\right)\right)=\sigma_{y}{}^{T}\left(t\right)(C_{2}{}^{-1}C_{1}{\dot{E}}_{y}\left(t\right)+(\rho_{2}z_{4}\left(t\right)\;+\frac{u_{z}\left(t\right)}{M}u_{y}\left(t\right)+\Delta_{y}\left(t\right)-{\ddot{y}}_{d}\left(t\right))+\alpha_{y}s_{y}{\left(t\right)}^{\frac{p_{y}}{q_{y}}})+{Q_{y}}_{psb}\left(t\right){{\dot{Q}}_{y}}_{psb}\left(t\right),\;{{\dot{V}}_{2}}_{z}\left(\sigma_{z}\left(t\right),{Q_{z}}_{a}\left(t\right)\right)=\sigma_{z}{}^{T}\left(t\right)(C_{2}{}^{-1}C_{1}{\dot{E}}_{z}\left(t\right)+(\rho_{3}z_{6}\left(t\right)-g+\frac{\cos\left(\phi \right)\cos\left(\theta \right)}{M}u_{z}\left(t\right)\;+\Delta_{z}\left(t\right)-{\ddot{z}}_{d}\left(t\right))+\alpha_{z}s_{z}{\left(t\right)}^{\frac{p_{z}}{q_{z}}})+{Q_{z}}_{psb}\left(t\right){{\dot{Q}}_{i}}_{psb}\left(t\right),\;{{\dot{V}}_{2}}_{\phi }\left(\sigma_{\phi }\left(t\right),{Q_{\phi }}_{a}\left(t\right)\right)=\sigma_{\phi }{}^{T}\left(t\right)(C_{2}{}^{-1}C_{1}{\dot{E}}_{\phi }\left(t\right)+(\rho_{4}z_{12}\left(t\right)z_{10}\left(t\right)+\rho_{5}z_{8}{}^{2}\left(t\right)\;+\rho_{6}\bar{\Omega }z_{10}\left(t\right)+\varrho_{1}u_{\phi }\left(t\right)+\Delta_{\phi }\left(t\right)-{\ddot{\phi }}_{d}\left(t\right))\;+\alpha_{\phi }s_{\phi }{\left(t\right)}^{\frac{p_{\phi }}{q_{\phi }}})+{Q_{\phi }}_{psb}\left(t\right){{\dot{Q}}_{\phi }}_{psb}\left(t\right),\;{{\dot{V}}_{2}}_{\theta }\left(\sigma_{\theta }\left(t\right),{Q_{\theta }}_{a}\left(t\right)\right)=\sigma_{\theta }{}^{T}\left(t\right)(C_{2}{}^{-1}C_{1}{\dot{E}}_{\phi }\left(t\right)+(\rho_{7}\dot{\psi }\left(t\right)\dot{\phi }\left(t\right)+\rho_{8}{\dot{\theta }}^{2}\left(t\right)\;+\rho_{9}\bar{\Omega }\dot{\phi }\left(t\right)+\varrho_{2}u_{\theta }\left(t\right)+\Delta_{\theta }\left(t\right)-{\ddot{\theta }}_{d}\left(t\right))+\alpha_{\phi }s_{\phi }{\left(t\right)}^{\frac{p_{\phi }}{q_{\phi }}})+{Q_{\theta }}_{psb}\left(t\right){{\dot{Q}}_{\theta }}_{psb}\left(t\right),\;{{\dot{V}}_{2}}_{\psi }\left(\sigma_{\psi }\left(t\right),{Q_{\psi }}_{a}\left(t\right)\right)=\sigma_{\psi }{}^{T}\left(t\right)(C_{2}{}^{-1}C_{1}{\dot{E}}_{\psi }\left(t\right)+(\rho_{10}z_{8}\left(t\right)z_{10}\left(t\right)+\rho_{11}z_{12}{}^{2}\left(t\right)\;+\varrho_{3}u_{\psi }\left(t\right)+\Delta_{\psi }\left(t\right)-{\ddot{\psi }}_{d}\left(t\right))+\alpha_{\psi }s_{\psi }{\left(t\right)}^{\frac{p_{\psi }}{q_{\psi }}})+{Q_{\psi }}_{psb}\left(t\right){{\dot{Q}}_{\psi }}_{psb}\left(t\right).$$

Substituting the equivalent controller (14) and discontinuous controller (27) into the above equation, we obtain:

(40) $${{\dot{V}}_{3}}_{i}\left(\sigma_{i}\left(t\right),{Q_{i}}_{psb}\left(t\right)\right)=-\sigma_{i}{}^{T}\left(t\right)\left(\left({Q_{i}}_{psb}\left(t\right)+\mu_{i}\right)\mathrm{sgn}(\sigma_{i}\left(t\right)\right)-\Delta_{i}\left(t\right))+{Q_{i}}_{psb}\left(t\right){{\dot{Q}}_{i}}_{psb}\left(t\right)\;\leq -\mu_{i}\left|\sigma_{i}\left(t\right)\right|+\left|\sigma_{i}\left(t\right)\right|\left|\Delta_{i}\left(t\right)\right|-{Q_{i}}_{psb}\left(t\right)\left|\sigma_{i}\left(t\right)\right|+{Q_{\psi }}_{psb}\left(t\right){{\dot{Q}}_{\psi }}_{psb}\left(t\right)\;\leq -\left({Q_{i}}_{psb}\left(t\right)-\left|\Delta_{i}\left(t\right)\right|\right)\left|\sigma_{i}\left(t\right)\right|+{Q_{i}}_{psb}\left(t\right)\frac{\varepsilon_{i}}{{\left(\varepsilon_{i}-\left|\sigma_{i}\left(t\right)\right|\right)}^{2}}\mathrm{sgn}(\sigma_{i}\left(t\right)){\dot{\sigma }}_{i}\left(t\right)\;\leq -\left({Q_{i}}_{psb}\left(t\right)-\left|\Delta_{i}\left(t\right)\right|\right)\left|\sigma_{i}\left(t\right)\right|+{Q_{i}}_{psb}\left(t\right)\frac{\varepsilon_{i}}{{\left(\varepsilon_{i}-\left|\sigma_{i}\left(t\right)\right|\right)}^{2}}\mathrm{sgn}(\sigma_{i}\left(t\right))\;\left(-\left({Q_{i}}_{psb}\left(t\right)+\mu_{i}\right)\mathrm{sgn}(\sigma_{i}\left(t\right))+\Delta_{i}\left(t\right)\right)\;\leq -\left({Q_{i}}_{psb}\left(t\right)-\left|\Delta_{i}\left(t\right)\right|\right)\left|\sigma_{i}\left(t\right)\right|-\frac{\varepsilon_{i}}{{\left(\varepsilon_{i}-\left|\sigma_{i}\left(t\right)\right|\right)}^{2}}\left({Q_{i}}_{psb}\left(t\right)-\left|\Delta_{i}\left(t\right)\right|\right){Q_{i}}_{psb}\left(t\right),$$

where, because ${Q_{i}}_{psb}>\left|\Delta_{i}\left(t\right)\right|$ and $\frac{\varepsilon_{i}}{{\left(\varepsilon_{i}-\left|\sigma_{i}\left(t\right)\right|\right)}^{2}}>0$, we obtain:

(41) $${{\dot{V}}_{3}}_{i}\left(\sigma_{i}\left(t\right),{Q_{i}}_{psb}\left(t\right)\right)\leq -\sqrt{2}\left({Q_{i}}_{psb}\left(t\right)-\left|\Delta_{i}\left(t\right)\right|\right)\frac{\left|\sigma_{i}\left(t\right)\right|}{\sqrt{2}}-\frac{\sqrt{2}\varepsilon_{i}}{{\left(\varepsilon_{i}-\left|\sigma_{i}\left(t\right)\right|\right)}^{2}}({Q_{i}}_{psb}\left(t\right)\;-\left|\Delta_{i}\left(t\right)\right|)\frac{{Q_{i}}_{psb}\left(t\right)}{\sqrt{2}}\;\leq -\sqrt{2}\left({Q_{i}}_{psb}\left(t\right)-\left|\Delta_{i}\left(t\right)\right|\right)\min\left\{1,\frac{\varepsilon_{i}}{{\left(\varepsilon_{i}-\left|\sigma_{i}\left(t\right)\right|\right)}^{2}}\right\}\left(\frac{\left|\sigma_{i}\left(t\right)\right|}{\sqrt{2}}+\frac{{Q_{i}}_{psb}\left(t\right)}{\sqrt{2}}\right)\;\leq -\Omega_{i}V_{3}{{}_{i}}^{\frac{1}{2}}\left(\sigma_{i}\left(t\right),{Q_{i}}_{psb}\left(t\right)\right)$$

where $\Omega_{i}=\sqrt{2}\left(Q_{i}{}_{psb}\left(t\right)-\left|\Delta_{i}\left(t\right)\right|\right)\min\left\{1,,\frac{\varepsilon_{i}}{{\left(\varepsilon_{i}-\left|\sigma_{i}\left(t\right)\right|\right)}^{2}}\right\}$. Then, the proof is completed. □

**Figure 1:**
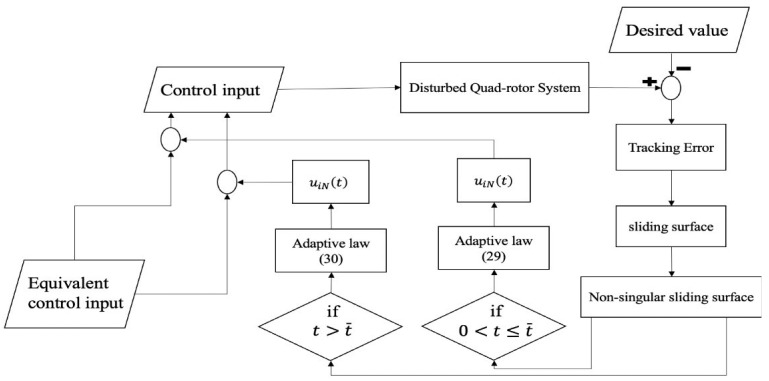
Block diagram of the barrier function based-adaptive non-singular TSMC.

In Figure 1, a flowchart of the designed control method using the barrier function adaptive non-singular TSMC is shown to provide an understanding of the control process.

**Remark** **3** **([37]).** *In order to overcome the chattering problem in the sliding mode control method, instead of the discontinuous function* $\mathrm{sgn}(\sigma_{i}\left(t\right))\;\left(\forall i=x,y,z,\phi ,\theta ,\psi \right)$ *, the hyperbolic tangent function* $\tanh\left(\frac{\sigma_{i}\left(t\right)}{\hbar_{i}}\right)$ *is used, where* ${\hbar_{i}}^{\prime }s$ *are the boundary layer thickness coefficients.*

**Remark** **4** **([38]).** *For implementation of the proposed method on the quad-rotor system, an outdoor environment based on the custom-built UAV platform can be applied for the conduction of flight trials. The required hardware and software, such as a mission planner using a Ground Control Station (GCS), flight controller, and an on-board computer, are used. Hence, the control signals can be received by an on-board computer in order to achieve the desired trajectory. Navigation of the quad-rotor is accomplished by sending the control signals of the attitude and altitude to the system motors.*

## 5. Simulation Results

In this section, the simulation outcomes using the barrier function-based adaptive non-singular TSMC approach are depicted in two different subsections. The two subsections are different with respect to the matched disturbances, such that in section *A*, the simulations results are shown without an abrupt change while in section *B*, an abrupt increase in the magnitude of the matched disturbances is examined. The parameters of the dynamic model of the quad-rotor system are shown in Table 2. In order to show the desired tracking of the attitude/position of the quad-rotor system, the desired vectors for the position/attitude of the quad-rotor are considered as $\left[x_{d}\left(t\right),y_{d}\left(t\right),z_{d}\left(t\right)]=[0.25,0.25,0.5\right]$ and $\left[\phi_{d}\left(t\right),\theta_{d}\left(t\right),\psi_{d}\left(t\right)\right]=\left[0.5\left(\sin\left(t+2\right)+\cos\left(t+2\right)\right),0.5\sin\left(t+2\right),\frac{\pi }{5}\right]$, respectively. In addition, the control parameters are obtained by trial and error and are shown in Table 3.

### 5.1. Simulation Results of the Barrier Function-Based Adaptive Non-Singular TSMC Method

**Figure 2:**
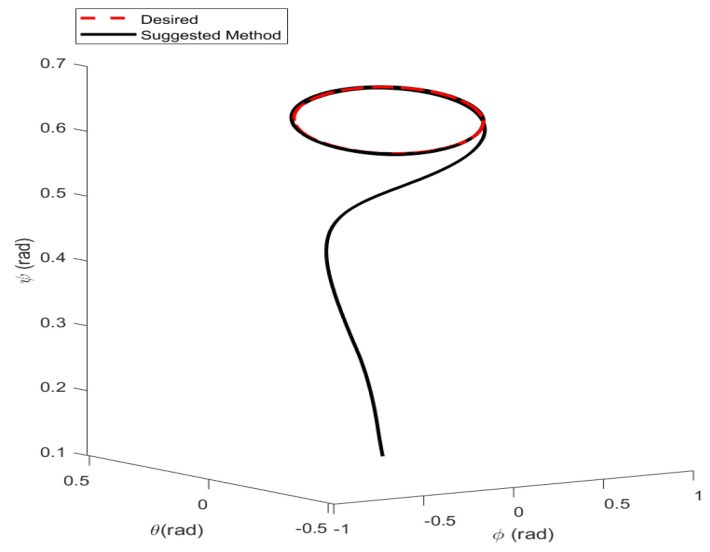
Three-dimensional schematic of attitude tracking of the quad-rotor using the barrier function based-adaptive non-singular TSMC.

**Figure 3:**
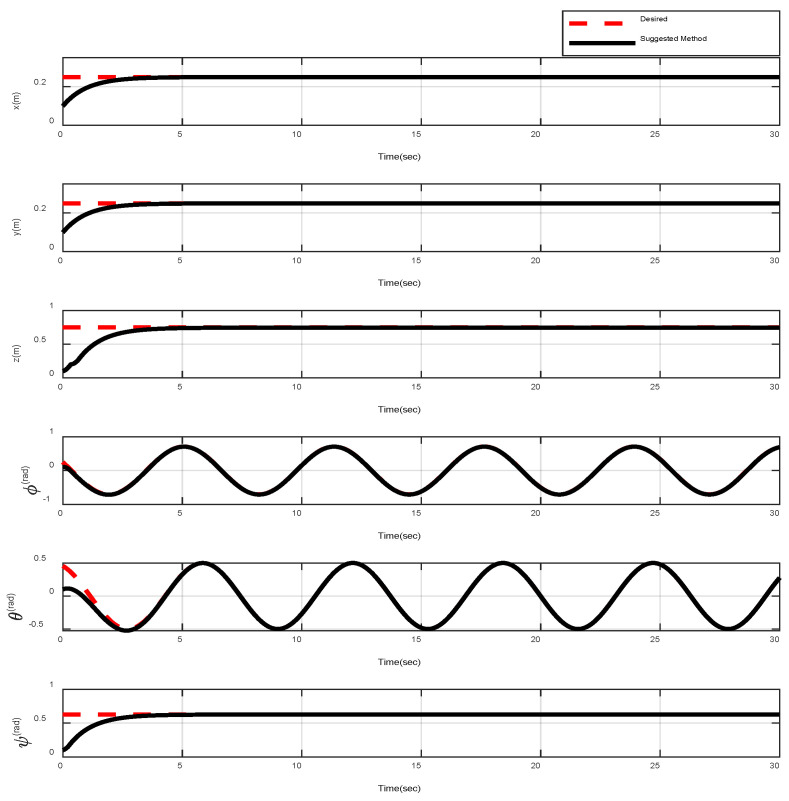
Position and attitude tracking of the quad-rotor using the barrier function-based adaptive non-singular TSMC method.

**Figure 4:**
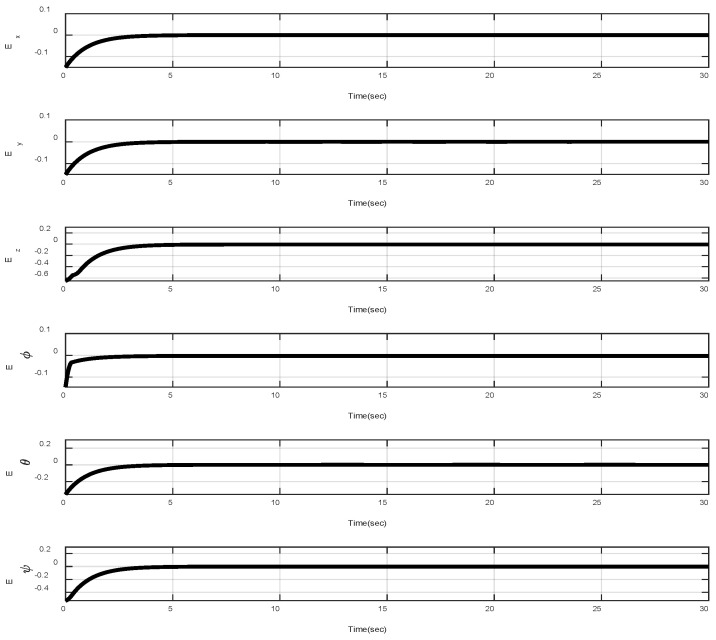
Trajectories of the position and tracking errors.

**Figure 5:**
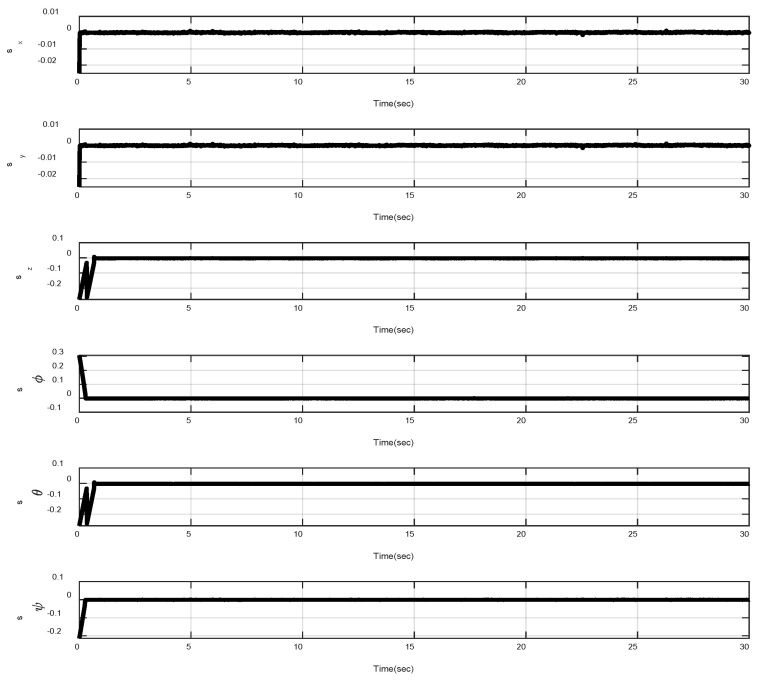
Trajectories of the linear sliding surfaces.

**Figure 6:**
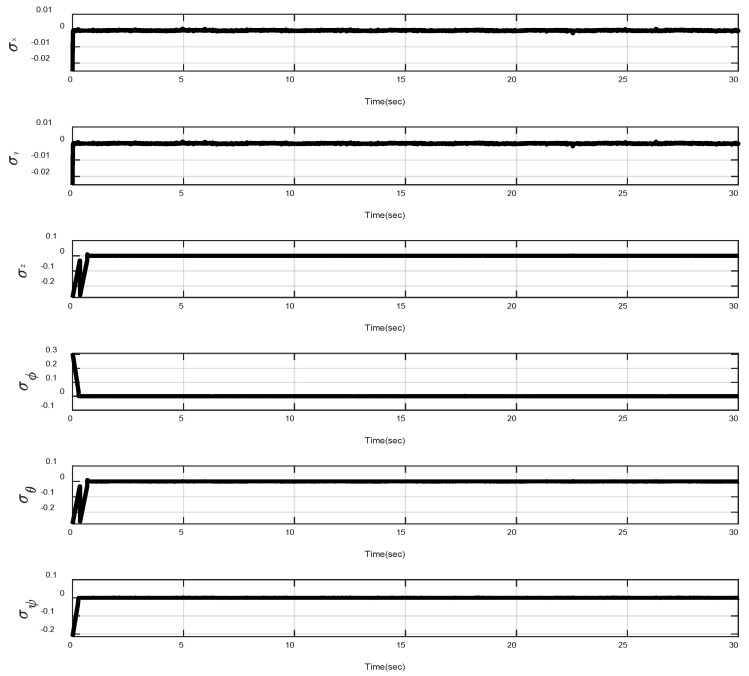
Trajectories of the non-singular TSMC surfaces.

**Figure 7:**
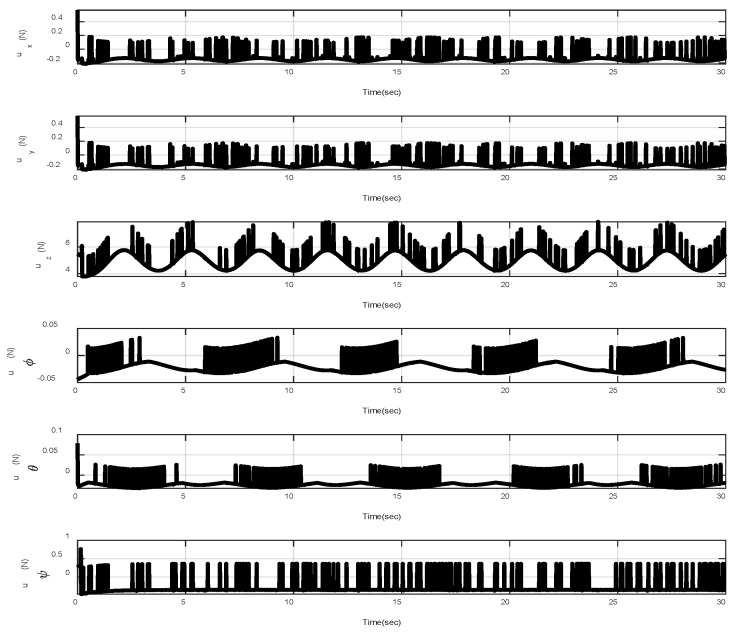
Control inputs.

**Figure 8:**
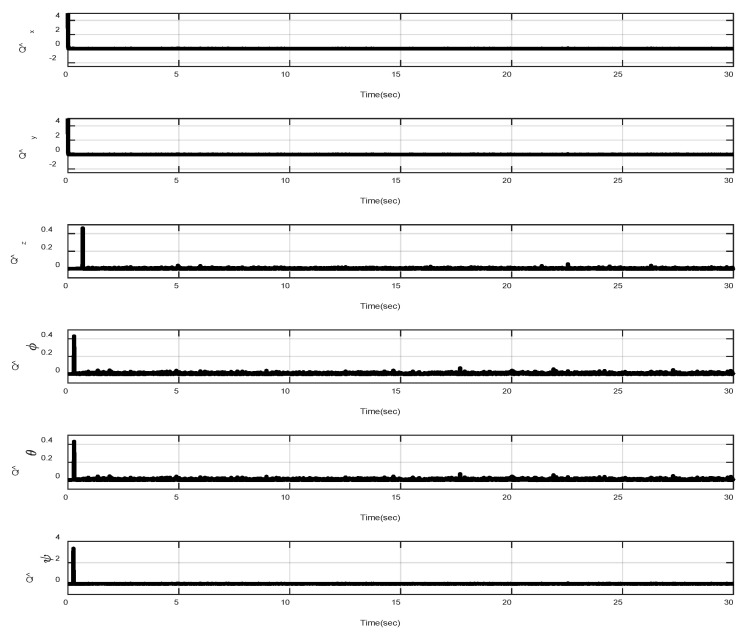
Trajectories of ${\hat{Q}}_{i}\left(t\right),\;\left(\forall i=x,y,z,\phi ,\theta ,\psi \right)$.

In the first example, the matched disturbances are considered as $\Delta_{i}\left(t\right)=1-0.1\sin\left(2t\right),\;\left(\forall i=x,y,z,\phi ,\theta ,\psi \right)$ and the simulation results are obtained. A three-dimensional schematic of the desired tracking of the attitude of the quad-rotor is shown in Figure 2. As one can observe from this figure, the quad-rotor system tracks the desired path when it starts from the initial point. The position/attitude tracking trajectories of the quad-rotor are shown in Figure 3. Therefore, the quad-rotor can track the desired position and attitude in finite time. Hence, the desired tracking is confirmed based on Figure 4, which shows the trajectories of the tracking error dynamics. The time histories of the linear and non-singular sliding surfaces are depicted in Figure 5 and Figure 6, respectively. Thus, the finite-time convergence of the recommended sliding manifolds is shown. The time trajectories of the control inputs using the barrier function-based adaptive non-singular TSMC approach are displayed in Figure 7. It can be seen that the amplitude of the control inputs is appropriate, and the control inputs are bounded. Finally, the time responses of the barrier function are displayed in Figure 8. It can be concluded that these signals are chattering-free, and they act in the limited and bounded range around zero.

### 5.2. Abrupt Change in Matched Disturbance

**Figure 9:**
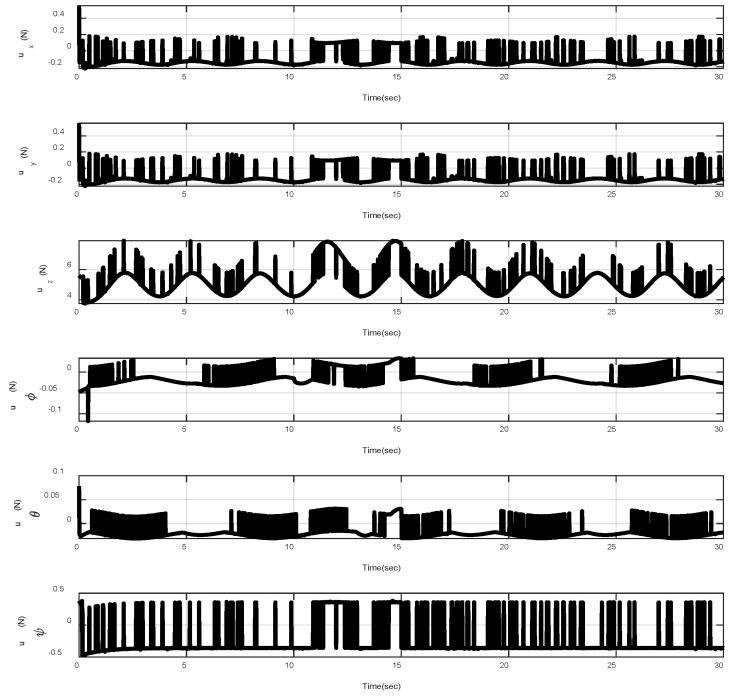
Control inputs in the presence of abrupt change.

**Figure 10:**
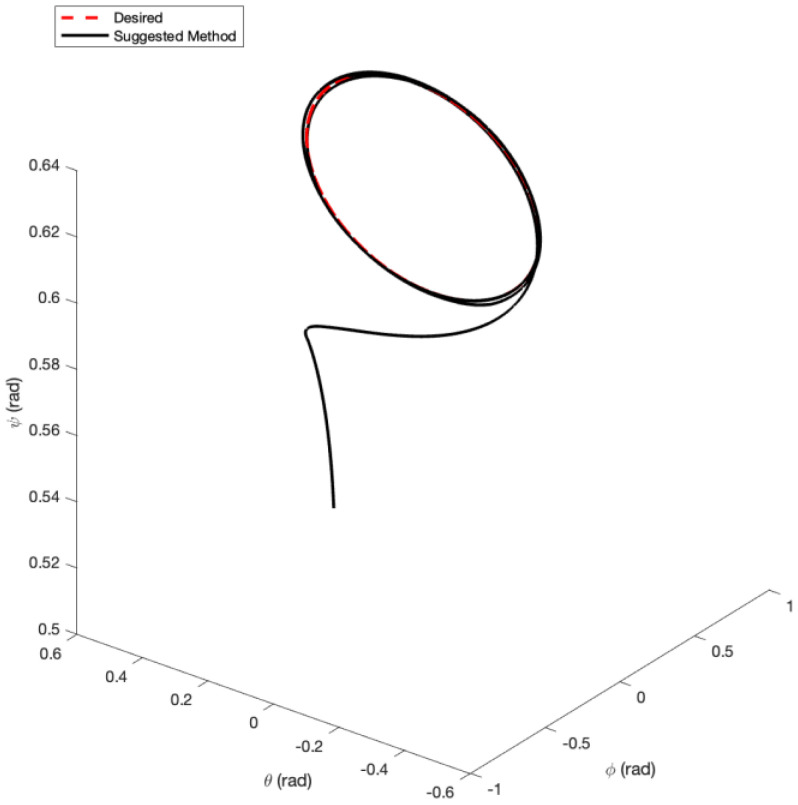
Three-dimensional schematic of attitude tracking of the quad-rotor using the barrier function-based adaptive non-singular TSMC under abrupt change.

**Figure 11:**
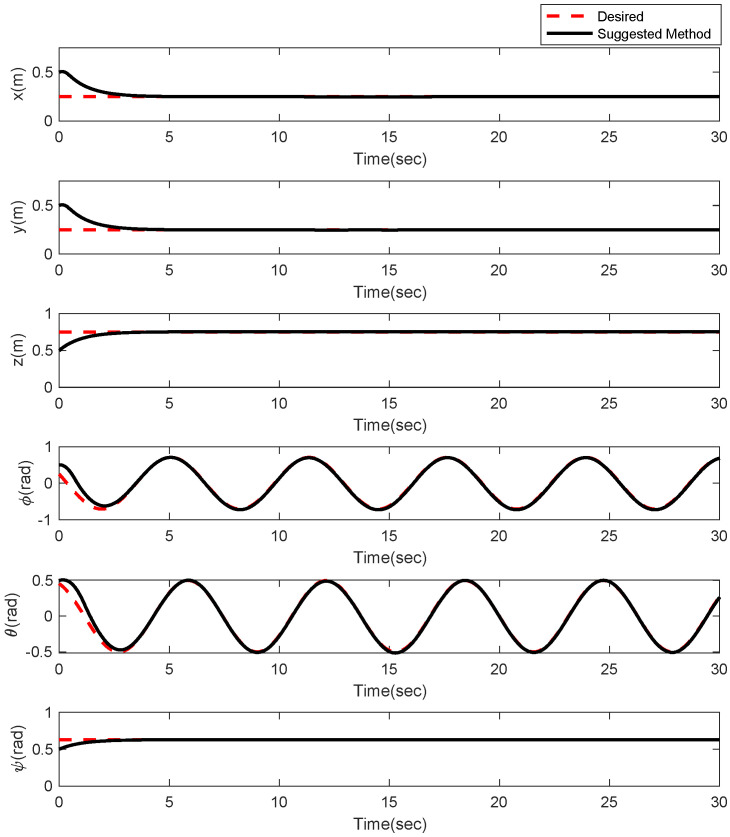
Position and attitude tracking of the quad-rotor using the barrier function-based adaptive non-singular TSMC method under abrupt change.

**Figure 12:**
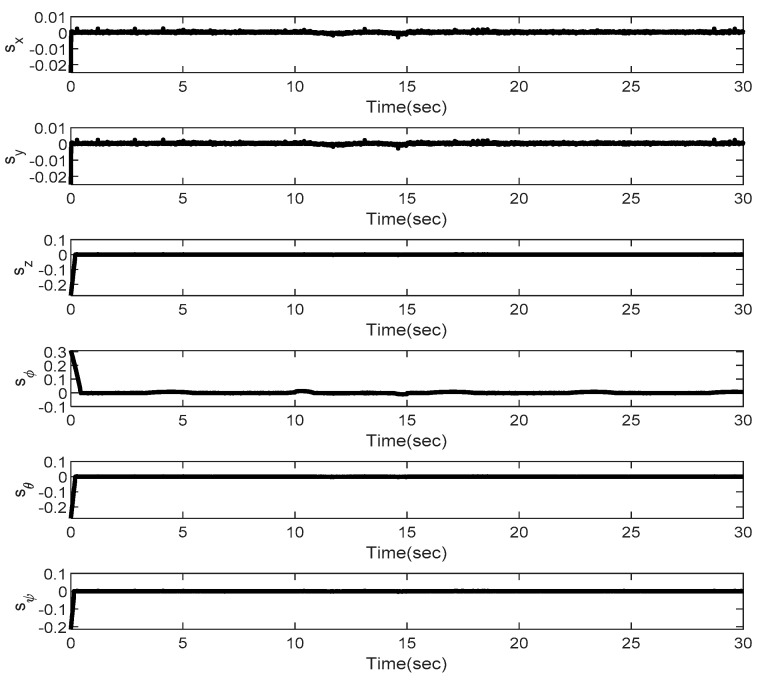
Trajectories of the linear sliding surfaces under abrupt change.

**Figure 13:**
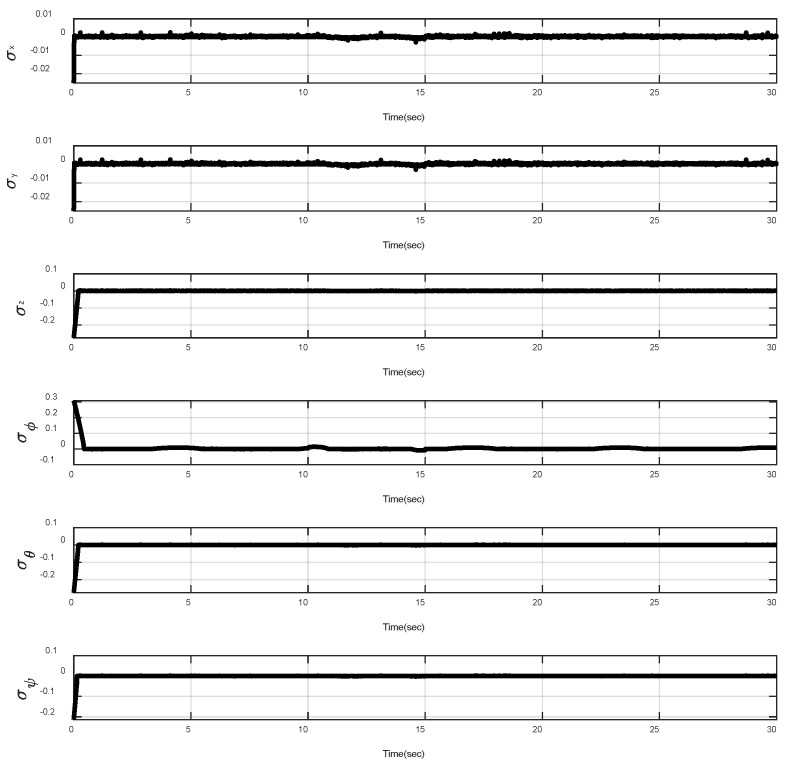
Trajectories of the non-singular sliding surfaces under abrupt change.

**Figure 14:**
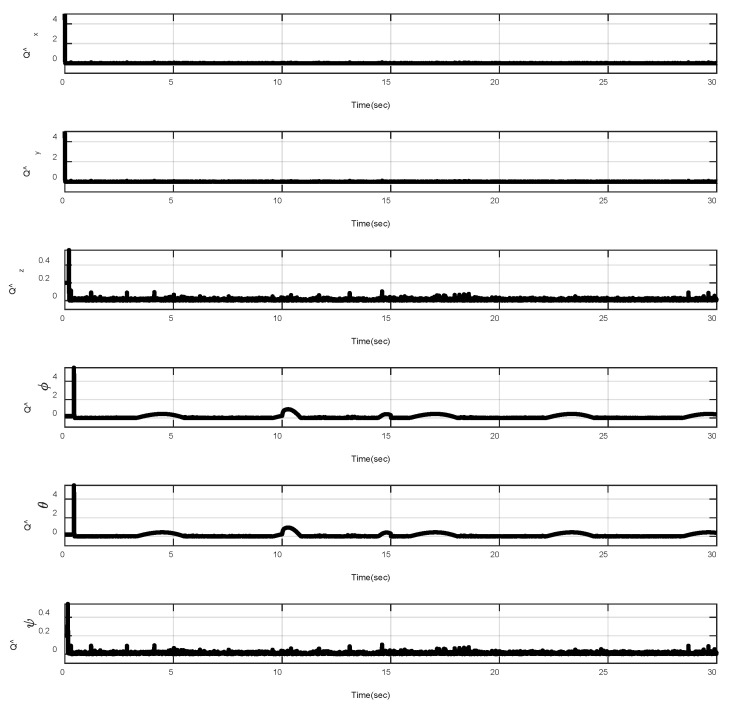
Trajectories of ${\hat{Q}}_{i}\left(t\right),\;\left(\forall i=x,y,z,\phi ,\theta ,\psi \right)$ under abrupt change.

In this example, the effect of an increase in the amplitude of disturbances in the considered interval is investigated. Thus, in the interval $\left[10,\;15\right]$, the matched disturbances are considered as $\Delta_{i}\left(t\right)=2.5\sin\left(2t\right),\;\left(\forall i=x,y,z,\phi ,\theta ,\psi \right)$. So, the amplitude of the *sin* function is 24-fold compared with the disturbance amplitude of the previous example. Furthermore, the effect of the abrupt change can be observed in the control inputs of Figure 9. After, the time trajectories of the 3-D desired tracking, position and attitude tracking, non-singular linear sliding surface, and barrier function are depicted in the presence of an abrupt change of the matched disturbances in Figure 10, Figure 11, Figure 12, Figure 13 and Figure 14, respectively. Based on these figures, it can be stated that the system states are stable and robust against an abrupt change of the matched disturbances. Therefore, the proficiency and efficiency of the proposed scheme are proved. In addition, initial condition is considered as $z(0)=[0.5,0.1,0.5,0.1,0.5,0.1,0.5,0.1,0.5,0.1,0.5,0.1{]}^{T}$.

## 6. Conclusions

In this study, the position and attitude dynamic equation of a 6DoF quad-rotor system were introduced. Then, to simplify the control strategy, the presented dynamic equation of the quad-rotor system was obtained in the state-space form with the appearance of matched disturbances. After, on the target of position/attitude tracking control of the quad-rotor, the sliding surfaces were defined based on the tracking error dynamics. In addition, new non-singular terminal sliding surfaces were proposed to achieve finite-time reachability of the linear sliding manifolds. In order to improve the robustness of the closed-loop system against matched disturbances, a non-singular adaptive terminal sliding mode control technique using the barrier function concept was designed. Finally, the simulation outcomes were provided to acknowledge the effectiveness of the suggested technique. As future research, two significant problems are noted, including consideration of the dynamical equation of the quad-rotor system with the existence of external disturbances, model uncertainties and input saturation, and simultaneous implementation of the proposed control method on the quadrotor UAVs in an experimental environment.

## Figures and Tables

**Table 1:** Parameters of the dynamical model of the quad-rotor [34,35,36].

Parameter	Description	Unit (*SI*)
$w_{i},\forall i=1,2,3,4$	Angular velocities	Rad/s
$I_{i},\forall i=xx,yy,zz$	Inertia respect to the x, y, z coordinates	N·m/rad/s^2^
${K_{fa}}_{i},\;\forall i=xx,yy,zz$	Aerodynamic fiction factors	N/rad/s
${K_{fd}}_{i},\forall i=xx,yy,zz$	Drag coefficients	N/rad/s
D	distance between rotation axes and center	m
M	Mass of quad-rotor	kg
$K_{p}$	lift power factor	N·m/rad/s
$J_{r}$	motor inertia	N·m/rad/s^2^
$C_{D}$	drag factors	N·m/rad/s

**Table 2:** Constants of dynamical model of the quad-rotor [34,35,36].

M=0.486	$C_{D}=3.2320\;\times$ 10^−2^	$\begin{array}{c} I_{i}=3.8278\times 10^{-5},\\ \forall i=xx,yy,zz \end{array}$
D=0.25	$J_{r}=2.8385\;\times$ 10^−5^	$\begin{array}{c} K_{fa}{}_{i}=K_{fd}{}_{i}=5.5670\times 10^{-4},\;\\ \forall i=xx,yy,zz \end{array}$

**Table 3:** Control parameters $\left(\forall i=x,y,z,\phi ,\theta ,\psi \right)$.

Variable	Value	Variable	Value
$z_{i}\left(0\right),\forall i=1,\ldots ,\;12$	0.1	$p_{i}$	5
$Q_{i}{}_{a}\left(0\right)$	0.2	$q_{i}$	3
$\ell_{i},\;$	0.03	$\mu_{i}$	1.5
$c_{1}=c_{2}$	0.5	$a_{i}$	0.1
$\varepsilon_{i},$	0.03		

## Data Availability

The data that support the findings of this study are available within the article.
